# Conditions prior to age 16 are associated with incident dementia status among older adults in the United States Health and Retirement

**DOI:** 10.1002/alz.089611

**Published:** 2025-01-09

**Authors:** Scarlet M Cockell, Herong Wang, Kelly S Benke, Erin B Ware, Kelly M. Bakulski

**Affiliations:** ^1^ University of Michigan, Ann Arbor, MI USA; ^2^ Johns Hopkins University, Baltimore, MD USA; ^3^ University of Michigan/ Insitute for Social Research, Ann Arbor, MI USA; ^4^ University of Michigan School of Public Health, Ann Arbor, MI USA

## Abstract

**Background:**

Dementia is a prevalent neurodegenerative disease with risk attributed to both genetic and environmental factors. Multiple factors contribute to the etiology of dementia, and the relevant exposure window of susceptibility is likely before symptom onset. Conditions and the environment in the early life period have not yet been comprehensively tested for association with later‐life dementia.

**Method:**

In the United States Health and Retirement Study (HRS) (n = 16,509; 2008‐2014 waves) 31 exposures occurring before age 16 were assessed via retrospective questionnaire among those with normal cognition (baseline measure). Incident dementia status (dementia, cognitively impaired non‐dementia, cognitively normal) was assessed over up to ten years of follow‐up. In this exposure‐wide association study, we tested for associations between exposures and cognitive status relative to normal cognition using parallel multivariable logistic models, adjusting sex, age at baseline, follow up time, race/ethnicity, personal education, and parental education.

**Result:**

In the analytic sample, participants were an average of 63 years of age at baseline. Over an average of 10 years of follow‐up, 5.3% of participants had incident dementia and 14.5% cognitive impairment. Depression before age 16 was associated with 1.71 (95% CI: 1.28, 2.26) times higher odds of incident cognitive impairment, relative to those with normal cognition. Headaches or migraines before age 16 were associated with 1.63 (95% CI: 1.18, 2.22) times higher odds of cognitive impairment, relative to normal cognition. Learning problems before age 16 were associated with 1.75 (95% CI:1.05, 2.79) times higher odds of cognitive impairment, relative to normal cognition. Self‐rated health as a child of “fair” (1.856 95% CI: 1.27, 2.64) and “poor” (3.39, 95% CI: 1.91, 5.82) were associated with odds of developing dementia, relative to self‐rated “good” childhood health.

**Conclusion:**

Conditions and exposures originating in early life may be important risk factors for later onset of cognitive impairment or dementia, extending the relevant window of susceptibility. Longitudinal research may be subject to challenges related to recall of information and differential survival and follow‐up studies are needed.

